# A phase I trial of S-1 with concurrent radiotherapy for locally advanced pancreatic cancer

**DOI:** 10.1038/sj.bjc.6603788

**Published:** 2007-05-29

**Authors:** M Ikeda, T Okusaka, Y Ito, H Ueno, C Morizane, J Furuse, H Ishii, M Kawashima, Y Kagami, H Ikeda

**Affiliations:** 1 Hepatobiliary and Pancreatic Oncology Division, National Cancer Center Hospital, Tokyo, Japan; 2 Radiation Oncology Division, National Cancer Center Hospital, Tokyo, Japan; 3 Hepatobiliary and Pancreatic Medical Oncology Division, National Cancer Center Hospital East, Chiba, Japan; 4 Radiation Oncology Division, National Cancer Center Hospital East, Chiba, Japan

**Keywords:** pancreatic cancer, chemoradiotherapy, radiosensitizer, S-1, CA19-9

## Abstract

This study investigated the maximum tolerated dose of S-1 based on the frequency of its dose-limiting toxicities (DLT) with concurrent radiotherapy in patients with locally advanced pancreatic cancer. S-1 was administered orally at escalating doses from 50 to 80 mg m^−2^ b.i.d. on the day of irradiation during radiotherapy. Radiation therapy was delivered through four fields as a total dose of 50.4 Gy in 28 fractions over 5.5 weeks, and no prophylactic nodal irradiation was given. Twenty-one patients (50 three; 60 five; 70 six; 80 mg m^−2^ seven patients) were enrolled in this trial. At a dose of 70 mg m^−2^ S-1, two of six patients demonstrated DLT involving grade 3 nausea and vomiting and grade 3 haemorrhagic gastritis, whereas no patients at doses other than 70 mg m^−2^ demonstrated any sign of DLT. Among the 21 enrolled patients, four (19.0%) showed a partial response. The median progression-free survival time and median survival time for the patients overall were 8.9 and 11.0 months, respectively. The recommended dose of S-1 therapy with concurrent radiotherapy is 80 mg m^−2^ day^−1^. A multi-institutional phase II trial of this regimen in patients with locally advanced pancreatic cancer is now underway.

Pancreatic cancer (PC) is one of the leading causes of cancer death worldwide. The prognosis of patients with this disease remains extremely poor, with a 5-year survival rate after diagnosis of less than 5%. Despite recent improvements in diagnostic techniques, PC is diagnosed at an advanced stage in most patients. Among these patients, roughly one-third is diagnosed as having locally advanced disease radiographically confined to the pancreas and surrounding tissues. In patients with locally advanced PC, the concurrent external-beam radiation therapy and 5-fluorouracil (5-FU) therapy has been shown to offer a survival benefit in comparison with radiotherapy alone (Moertel *et al*, 1969, 1981) or chemotherapy alone (Gastrointestinal Tumor Study Group, 1988). In an attempt to improve the efficacy of 5-FU with concurrent radiotherapy, various anticancer agents and radiation schedules are being examined in clinical trials, but no significant impact on survival has been accomplished. Because of these results, 5-FU with concurrent radiotherapy remains the predominant chemoradiotherapy for locally advanced PC in clinical use (Willett *et al*, 2005; Yip *et al*, 2006).

S-1 is a novel orally administered drug, which is a combination of tegafur, 5-chloro-2,4-dihydroxypyridine and oteracil potassium in a 1 : 0.4 : 1 molar concentration ratio. Tegafur is hydroxylated and converted to 5-FU by the hepatic microsomal enzymes. 5-Chloro-2,4-dihydroxypyridine is a competitive inhibitor of dihydropyrimidine dehydrogenase, which is involved in the degradation of 5-FU, and acts to maintain effective concentrations of 5-FU in plasma and tumour tissues. Oteracil potassium, a competitive inhibitor of orotate phosphoribosyltransferase, inhibits the phosphorylation of 5-FU in the gastrointestinal tract, reducing the serious gastrointestinal toxicity associated with 5-FU (Shirasaka *et al*, 1996a). In athymic nude rats, S-1 has been shown to result in retention of a higher and more prolonged concentration of 5-FU in plasma and tumour tissues in comparison with 5-FU and uracil/tegafur (Shirasaka *et al*, 1996b). The antitumour effect of S-1 has already been demonstrated in a variety of solid tumours, including advanced gastric cancer (Sakata *et al*, 1998), colorectal cancer (Ohtsu *et al*, 2000), non-small-cell lung cancer (Kawahara *et al*, 2001), and head and neck cancer (Inuyama *et al*, 2001). In patients with metastatic PC, a recent early phase II study has demonstrated a response rate of 21% (Ueno *et al*, 2005), and a more favourable tumour response (response rate: 38%) and survival (median: 8.8 months) have been reported in a multi-institutional late phase II trial of S-1 (Furuse *et al*, 2005).

Thus, S-1 has promising antitumour activity against advanced PC, and is much more convenient to administer than intravenous 5-FU infusion, as it is taken orally. Concurrent radiotherapy along with S-1 therapy as an alternative to 5-FU infusion may result in more efficient treatment and improve the quality of life of patients. Therefore, we conducted a phase I trial to determine the maximum tolerated dose of S-1 with concurrent radiotherapy based on the frequency of dose-limiting toxicities (DLT) in patients with locally advanced PC.

## PATIENTS AND METHODS

### Eligibility

Patients eligible for study entry had histologically or cytologically confirmed locally advanced nonresectable PC. Eligibility criteria were age 20–74 years; Karnofsky performance status 70–100 points; no evidence of distant metastasis; adequate oral intake; estimated life expectancy ⩾12 weeks after study entry; no earlier treatment for PC; adequate haematological function (haemoglobin ⩾10 g dl^−1^, leucocytes ⩾4000 mm^−3^, neutrophils ⩾2000 mm^−3^ and platelets ⩾100 000 mm^−3^); adequate hepatic function (serum total bilirubin ⩽2.0 times upper normal limit (UNL); serum albumin ⩾3.0 g dl^−1^ and serum transaminases (aspartate aminotransferase (AST)/alanine aminotransferase (ALT)) ⩽2.5 times UNL or ⩽5 times UNL if biliary drainage present); adequate renal function (serum creatinine ⩽1.0 mg dl^−1^); written informed consent.

The exclusion criteria were watery diarrhoea; pleural effusion or ascites; active infection; active gastroduodenal ulcer; severe complication such as heart disease or renal disease; mental disorder; history of drug hypersensitivity; active concomitant malignancy; pregnant and lactating females; females of childbearing age unless using effective contraception.

Ultrasonography, multidetector row-computed tomography of the abdomen and chest X-ray were performed for pretreatment staging to assess the local extension of the tumour and exclude the presence of distant metastasis. The computed tomography-based criteria for tumour nonresectability included tumour encasement of the celiac trunk, common hepatic artery, superior mesenteric artery or bilateral invasion of the portal vein. All patients with obstructive jaundice underwent percutaneous transhepatic or endoscopic retrograde biliary drainage before treatment. This phase I study was approved by the Institutional Review Board of the National Cancer Center and conducted in accordance with the Declaration of Helsinki Principles.

### Treatment schedule

This was an open-label, two-institutional and single-arm phase I study that was performed on an in-patient basis. Radiotherapy was administered by 10 or 25 MV photons using three-dimensional treatment planning. A total dose of 50.4 Gy was delivered in 28 fractions over 5.5 weeks. The clinical target volume (CTV) included only the gross primary tumour and nodal involvement enlarged over 10 mm detected by computed tomography. Elective nodal irradiation was not used. The planning target volume was defined as CTV plus a 10 mm margin in the lateral direction and 10–20 mm margin in the craniocaudal direction to account for respiratory organ motion and daily set-up error. The four-field technique (anterior, posterior and opposed lateral fields) was used. There was no field reduction. The spinal cord dose was maintained below 45 Gy. The dose received by ⩾50% of the liver was limited to ⩽30 Gy, and that received by ⩾50% of both kidneys was limited to ⩽20 Gy.

S-1 was administered orally twice daily after breakfast and dinner on the day of irradiation (Monday to Friday) during radiotherapy. The initial dose of S-1 was 50 mg m^−2^ day^−1^, and the dose was escalated to 80 mg m^−2^ day^−1^ in increments of 10 mg m^−2^ day^−1^ (Table 1). The calculated S-1 dose was rounded down to the nearest 60, 80, 100 or 120 mg. S-1 at 50 mg m^−2^ day^−1^ is reported to be almost equivalent to 200 mg m^−2^ day^−1^ intravenously 5-FU (Hirata *et al*, 1999), which has been used in protracted 5-FU infusion with concurrent radiotherapy for locally advanced PC at our institutions (Ishii *et al* 1997). S-1 at 80 mg m^−2^ day^−1^ is the standard dose used as a single agent for systemic therapy (Furuse *et al*, 2005; Ueno *et al*, 2005). Patients maintained a daily journal to record their intake of S-1 and any signs or symptoms that they experienced.

Patient cohorts had a minimum of three patients at each dose level. If no DLT was observed in the initial three patients, the dosage was escalated in successive cohorts. If DLT was observed in one or two of the initial three patients, three additional patients were evaluated at that dose level. If only one or two of six patients experienced DLT, dose escalation was continued. However, if three or more patients experienced DLT at a given dose level, then the previous dose level was considered as the maximum tolerated dose. Dose-limiting toxicities was defined as the following manifestations of toxicity observed until completion of chemoradiotherapy: grade 3 leucocytopenia and/or neutropenia with a fever ⩾38°C lasting 3 days or more, grade 3 leucocytopenia and/or neutropenia with infection, grade 4 leucocytopenia and/or neutropenia lasting 3 days or more, grade 4 leucocytopenia and/or neutropenia requiring haematopoietic colony-stimulating factors, platelets <25 000 mm^−3^, grade 3 thrombocytopenia requiring transfusion, serum AST/ALT ⩾10 times UNL, grade 3 or 4 nonhaematological toxicities excluding nausea, vomiting, anorexia, fatigue, constipation, hyperglycaemia, and abnormality of sodium, potassium, and calcium or treatment interruption for longer than 12 days.

When grade 3 or greater haematological toxicity, total bilirubin level 2.0–3.0 times UNL, serum AST/ALT 5.0–10.0 times UNL, grade 3 vomiting and/or grade 2 nonhaematological toxicity excluding nausea, vomiting, anorexia, fatigue, constipation, alopecia and pigmentation change, were observed, radiotherapy and S-1 administration was suspended. Treatment was resumed when the toxicities were resolved by one grade or more, compared with these suspension criteria. Dose modification was not performed in this study. When DLT or tumour progression was observed during chemoradiotherapy, this treatment was discontinued. After this treatment, the patients were allowed to receive another anticancer treatment at their physician's discretion.

### Toxicity and response evaluation

The primary end point of this trial was to evaluate the frequency of DLT, and the secondary end point was to evaluate the potential antitumour activity. Treatment-related toxicities were assessed using the National Cancer Institute Common Toxicity Criteria version 2.0. During this treatment, complete blood count with differentials, serum chemistry and urinalysis were carried out at least once a week. Tumor response was evaluated at the completion of chemoradiotherapy and every 8 weeks thereafter until tumour progression, according to the Japan Society for Cancer Therapy criteria (Japan Society for Cancer Therapy, 1993) as follows: a complete response was defined as the disappearance of all clinical evidence of the tumour for a minimum of 4 weeks. A partial response was defined as a 50% or greater reduction in the sum of the products of two perpendicular diameters of all measurable lesions for a minimum of 4 weeks. A minor response was defined as a 25% or greater reduction and less than 50% in the sum of the products of two perpendicular diameters of all measurable lesions for a minimum of 4 weeks or a 50% or greater reduction in the sum of the products of two perpendicular diameters of all measurable lesions lasting less than 4 weeks. No change was defined as a reduction of less than 25% or a less than 25% increase in the sum of the products of two perpendicular diameters of all lesions for a minimum of 4 weeks. Progressive disease was defined as an increase of 25% or more in the sum of the products of two perpendicular diameters of all lesions, or the appearance of any new lesion. Progression-free survival time was defined as the time from the date of initial treatment to the first documentation of progression or death. Overall survival was measured from the date of initial treatment to date of death or the date of the last follow-up. Progression-free and overall survival times were calculated by the Kaplan–Meier method. Serum carcinoembryonic antigen (CEA) levels and serum carbohydrate antigen 19-9 (CA19-9) levels were measured at least every 8 weeks by a radioimmunometric assay using the Centocor radioimmunoassay kit (Centocor Inc., Malvern, PA, USA).

## RESULTS

### Patient characteristics

Twenty-one patients were enrolled in this study from May 2004 and November 2005 at the National Cancer Center Hospital, Tokyo, and the National Cancer Center Hospital East, Kashiwa, Chiba, Japan. The characteristics of the patients are listed in Table 2. The median age was 59 years (range: 51–74). Karnofsky performance status was 100 in 12 patients (57%), 90 in 8 (38%) and 80 in one (5%). The median maximum tumour size was 37 mm (range: 25–60), and the median planning target volume was 265 cm^3^ (range: 153–408). The causes of the unresectable PCs were invasion of the celiac trunk in nine patients, invasion of the superior mesenteric artery in eight patients and invasion of both regions in four patients. Patients were treated with S-1 and concurrent radiation over four dose levels, as listed in Table 1. After completion of chemoradiotherapy, 20 patients (95%) received gemcitabine alone for their cancer until disease progression, and one patient received the other treatment at another hospital.

### Toxicity

The toxicities observed in the 21 enrolled patients are listed in Table 3. With regard to overall haematological toxicity, grade 3 neutropenia was observed in only one patient at the dose of level 1, and other grades 3–4 toxicities were not observed. For nonhaematological toxicity, grade 3 anorexia and nausea (three patients), grade 3 vomiting (one patient) and grade 3 haemorrhagic gastritis (one patient) occurred at level 3, and grade 3 AST elevation was observed in a patient at level 4. As a late toxicity, duodenal ulcer with epigastralgia was observed in one patient at level 3 (S-1 70 mg m^−2^) 8 months after chemoradiotherapy, requiring embolisation of the gastroduodenal artery to treat severe bleeding from the ulcer and a 2-month hospital stay. No other grades 3–4 nonhaematological toxicities or treatment-related deaths occurred in this study. Treatment was suspended in four patients (level 2, one; level 3, two; level 4, one patient) because of obstructive jaundice (two patients) or grade 3 anorexia (two patients); the durations of S-1 dose withholding were 3, 12, 2 and 13 days, respectively. One patient with grade 3 anorexia (level 3) was unable to resume this treatment. The compliance rate of the patients taking S-1 was as high as 99% (1170/1176 doses).

There was no occurrence of DLT at the dose of levels 1 or 2, but two of six patients who received a level 3 dose experienced DLT; one of these patients required suspension of treatment for more than 12 days due to grade 3 anorexia, nausea and vomiting after the third fraction of chemoradiotherapy, and a second developed grade 3 haemorrhagic gastritis after completion of 13 fractions. However, no DLT at a dose of level 4 was observed, and S-1 at 80 mg m^−2^ with concurrent radiotherapy was considered to be well-tolerated.

Five patients (level 2, two; level 3, two; level 4, one) of the 21 who were enrolled had to abandon this treatment. Two patients at level 2 developed massive ascites and infarction of the cerebellum, respectively, during chemoradiotherapy. The cause of the massive ascites was disease progression, as cancer cells were confirmed in the ascitic fluid. The cerebellar infarction was considered to have been unrelated to the treatment, because the patient had a history of the same problem. Two patients at level 3 had to discontinue the treatment because of DLT according to the protocol, and one patient at level 4 decided to stop the treatment, despite lack of severe toxicity, at her own request.

### Efficacy

**Figure 1:**
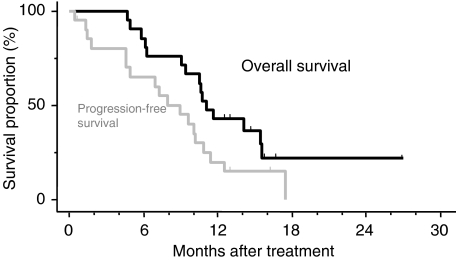
Overall survival and progression-free survival curves of 21 patients who received S-1 with concurrent radiotherapy for locally advanced pancreatic cancer. Tick marks indicate censored cases.

All the patients were included in the response evaluation. Four patients (levels 1 and 2, 0; level 3, one; level 4, three) achieved a partial response, giving an overall response rate of 19% (95% confidence interval, 5–42%). Four patients (19%) showed a minor response, and nine (43%) and three patients (14%) had no change and progressive disease, respectively. Tumor response could not be evaluated in one patient (5%), because she was transferred to another hospital to seek some other treatment for her PC. None of the patients' conditions improved to resectable or operable diseases after the completion of treatment. After the start of chemoradiotherapy, the serum CA19-9 level was reduced by more than 50% compared to the pretreatment level in 14 (88%) of 16 patients who had shown a pretreatment level of 100 U/ml or greater, and the serum CEA level was reduced by more than 50% relative to the pretreatment level in three (100%) of three patients who had a pretreatment level of 10 ng ml^−1^ or greater. Eighteen of the 21 patients had disease progression at the time of analysis. The pattern of disease progression was distant metastases in 11 (52%), deterioration of general condition in five (24%) and locoregional recurrence in two patients (10%). The median progression-free survival time for all patients was 8.9 months (Figure 1). At the time of analysis, 13 patients had died due to tumour progression. The median survival time and 1-year survival rate for patients as a whole were 11.0 months and 42.9%, respectively (Figure 1).

## DISCUSSION

On the basis of results of previous randomised controlled trials (Moertel *et al*, 1969, 1981; Gastrointestinal Tumor Study Group, 1988), the combination of 5-FU therapy and radiotherapy has become a frequently employed treatment for locally advanced PC (Willett *et al*, 2005; Yip *et al*, 2006). Because of the modest survival benefit of 5-FU-based chemoradiotherapy, numerous investigators are pursuing phase I and II trials of radiotherapy with new chemotherapeutic agents such as gemcitabine, paclitaxel, capecitabine, bevacizumab, gefitinib and erlotinib (Blackstock *et al*, 2003; Okusaka *et al*, 2004; Rich *et al*, 2004; Crane *et al*, 2006; Czito *et al*, 2006). However, no marked improvement of survival has been observed. S-1 is an oral fluoropyrimidine derivative that has demonstrated excellent efficacy with mild toxicity in patients with metastatic PC (Furuse *et al*, 2005). It is considered to be beneficial because of its convenience of being administered by the oral route. In addition, combined S-1 and radiotherapy has been demonstrated to exert a synergistic effect against 5-FU-resistant cancer xenografts (Harada *et al*, 2004; Nakata *et al*, 2006). Therefore, a clinical trial of concurrent radiotherapy with S-1 therapy for locally advanced PC was designed to intensify the treatment efficacy and improve the convenience of administration.

In this study, a limited radiation field, of which the planning target volume included only the gross tumour volume without prophylactic nodal irradiation, was adopted to minimise the volume of normal tissue treated, because our retrospective study showed that a larger planning target volume for irradiation was the significant predictor of severe acute gastrointestinal toxicity in patients treated with chemoradiotherapy (Ito *et al*, 2006). A similar radiation field has been attempted in recent reported trials of chemoradiotherapy to decrease the degree of gastrointestinal toxicity (Muler *et al*, 2004; Crane *et al*, 2006). Gastrointestinal toxicities, such as anorexia, nausea and vomiting, are major troublesome adverse events during chemoradiotherapy, necessitating intravenous fluid infusion and sometimes discontinuation of chemoradiotherapy (Talamonti *et al*, 2000; Crane *et al*, 2002; McGinn and Zalupski, 2003; Okusaka *et al*, 2004). In the present study, some gastrointestinal toxicities were observed, but were easily managed. Moreover, the limited radiation field used in this study did not result in excess failures in the border of radiation field, because locoregional recurrence was observed in only two patients of this series.

In this study, DLT was observed in only two patients at level 3 (S-1 70 mg m^−2^). The DLT in the first patient was grade 3 anorexia, nausea and vomiting, requiring suspension of treatment for longer than 12 days, and the second DLT was grade 3 haemorrhagic gastritis. Other than DLT toxicity, acute grades 3–4 toxicities during chemoradiotherapy were observed in only three patients: grade 3 neutropenia, grade 3 anorexia and nausea, and grade 3 AST elevation in one patient each. As a late toxicity, duodenal ulcer was observed 8 months after treatment in one patient at level 3, but no other late toxicity occurred. Accordingly, S-1 at a daily dose of 80 mg m^−2^ (level 4) was considered to be well tolerated, and this dose was deemed recommendable.

In patients with locally advanced PC who are receiving chemoradiotherapy, it is important to enhance local control while simultaneously reducing the risk of distant metastases. In concurrent gemcitabine-based chemoradiotherapy, both full-dose gemcitabine and standard-dose radiotherapy are difficult to administer because of their associated toxicities (Crane *et al*, 2002; Blackstock *et al*, 2003; McGinn and Zalupski, 2003; Okusaka *et al*, 2004). In contrast, in the present trial, the combination of full-dose S-1 (80 mg m^−2^) and standard-dose radiotherapy (50.4 Gy/28 fractions) was easy to administer and had favourable toxicity profiles. Therefore, this regimen might have a dual benefit of counteracting systemic tumour spread as well as acting as a potent radiosensitizer for local control. With regard to the antitumour activity of this treatment, four (19%) of the 21 patients achieved a partial response, and the response rate at the recommended dose was 43% (3/7). The progression-free survival time (median: 8.9 months) and overall survival time (median: 11.0 months) were also favourable as a phase I trial. In this study, many patients (95%) received gemcitabine alone after completion of this regimen. Such adjuvant gemcitabine therapy might influence the efficacy of treatment, although the extent of its impact on tumour response and survival has not been fully elucidated in patients with locally advanced PC. Since both the efficacy and toxicity profile of this regimen appear to be attractive, a phase II trial is required to clarify the antitumour activity, survival and toxicity of S-1 80 mg m^−2^ day^−1^ with concurrent radiation therapy for locally advanced PC.

In conclusion, the recommended dose of S-1 with concurrent radiotherapy is 80 mg m^−2^ day^−1^ on the day of irradiation, and this regimen has a mild toxicity profile while delivering substantial antitumour activity for patients with locally advanced PC. Orally administered S-1 may offer an easy alternative to intravenous 5-FU without impairing the quality of life. A phase II trial of S-1 at the optimal dose of 80 mg m^−2^ day^−1^ with concurrent radiation in patients with locally advanced PC is now underway in a multi-institutional setting.

## Figures and Tables

**Table 1:** Dose schedules of S-1 with concurrent radiotherapy

**Dosage level**	**S-1 dose (mg m^−2^ day^−1^)**	**Number of patients**
1	50	3
2	60	5
3	70	6
4	80	7

**Table 2:** Patient characteristics

**Characteristics**	**Number of patients**	**%**
*Age (years)*		
Median	59	
Range	51–74	
		
*Gender*		
Male	9	43
Female	12	57
		
*Karnofsky performance status*		
100	12	57
90	8	38
80	1	5
		
*Tumour location*		
Head	13	62
Body-tail	8	38
		
*Maximum tumour size (mm)*		
Median	37	
Range	25–60	
		
*CEA (ng/ml)*		
Median	4.5	
Range	1.0–75.0	
		
*CA19-9 (U/ml)*		
Median	759	
Range	1–6,300	

CEA=carcinoembryonic antigen; CA19–9=carbohydrate antigen 19–9.

**Table 3:** Toxicity

	**Number of patients**											
	**Level 1 (*n*=3)**			**Level 2 (*n*=5)**			**Level 3 (*n*=6)**			**Level 4 (*n*=7)**		
Grade	1,2	3	4	1,2	3	4	1,2	3	4	1,2	3	4
Leucocytes	3	0	0	3	0	0	3	0	0	6	0	0
Neutrophiles	1	1	0	1	0	0	2	0	0	3	0	0
Haemoglobin	0	0	0	2	0	0	1	0	0	4	0	0
Platelets	0	0	0	1	0	0	1	0	0	2	0	0
Anorexia	2	0	0	3	0	0	1	3	0	5	0	0
Nausea	0	0	0	2	0	0	1	3	0	6	0	0
Vomiting	1	0	0	0	0	0	2	1	0	3	0	0
Diarrhoea	1	0	0	0	0	0	0	0	0	0	0	0
Mucositis	0	0	0	0	0	0	0	0	0	0	0	0
Fatigue	2	0	0	2	0	0	2	0	0	2	0	0
Gastritis	0	0	0	0	0	0	0	1	0	0	0	0
Duodenal ulcer	0	0	0	0	0	0	0	1	0	0	0	0
Bilirubin	1	0	0	0	0	0	0	0	0	0	0	0
Hypoalbuminaemia	1	0	0	1	0	0	3	0	0	5	0	0
AST	1	0	0	1	0	0	4	0	0	2	0	0
ALT	1	0	0	0	0	0	3	0	0	1	1	0
Alkaline phosphatase	0	0	0	0	0	0	1	0	0	2	0	0
Creatinine	0	0	0	0	0	0	1	0	0	0	0	0

AST=aspartate aminotransferase; ALT=alanine aminotransferase.
